# Artificial intelligence, extended reality, and emerging AI–XR integrations in medical education

**DOI:** 10.3389/fdgth.2025.1740557

**Published:** 2026-01-09

**Authors:** Talia Tene, Diego Fabián Vique López, Marlene Jacqueline García Veloz, Byron Stalin Rojas Oviedo, Richard Tene-Fernandez

**Affiliations:** 1 Department of Chemistry, Universidad Técnica Particular de Loja, Loja, Ecuador; 2 Facultad de Salud Pública, Escuela Superior Politécnica de Chimborazo (ESPOCH), Riobamba, Ecuador; 3 Facultad de Ciencias, Escuela Superior Politécnica de Chimborazo (ESPOCH), Riobamba, Ecuador; 4 Hospital Metropolitano de Quito, Quito, Ecuador

**Keywords:** artificial intelligence, ChatGPT, competency-based education, digital literacy, health education, prompt engineering, simulation

## Abstract

**Introduction:**

Artificial intelligence (AI) and extended reality (XR)—including virtual, augmented, and mixed reality—are increasingly adopted in health-professions education. However, the educational impact of AI, XR, and especially their combined use within integrated AI–XR ecosystems remains incompletely characterized.

**Objective:**

To synthesize empirical evidence on educational outcomes and implementation considerations for AI-, XR-, and combined AI–XR–based interventions in medical and health-professions education.

**Methods:**

Following PRISMA and PICO guidance, we searched three databases (Scopus, PubMed, IEEE Xplore) and screened records using predefined eligibility criteria targeting empirical evaluations in health-professions education. After deduplication (336 records removed) and two-stage screening, 13 studies published between 2019 and 2024 were included. Data were extracted on learner population, clinical domain, AI/XR modality, comparators, outcomes, and implementation factors, and narratively synthesized due to heterogeneity in designs and measures.

**Results:**

The 13 included studies involved undergraduate and postgraduate learners in areas such as procedural training, clinical decision-making, and communication skills. Only a minority explicitly integrated AI with XR within the same intervention; most evaluated AI-based or XR-based approaches in isolation. Across this mixed body of work, studies more often than not reported gains in at least one outcome—knowledge or skills performance, task accuracy, procedural time, or learner engagement—relative to conventional instruction, alongside generally high acceptability. Recurrent constraints included costs, technical reliability, usability, faculty readiness, digital literacy, and data privacy and ethics concerns.

**Conclusions:**

Current evidence on AI, XR, and emerging AI–XR integrations suggests promising but preliminary benefits for learning and performance. The small number of fully integrated AI–XR interventions and the methodological limitations of many primary studies substantially limit the certainty and generalizability of these findings. Future research should use more rigorous and standardized designs, explicitly compare AI-only, XR-only, and AI–XR hybrid approaches, and be coupled with faculty development, robust technical support, and alignment with competency-based assessment.

## 1 Introduction

Medical education is undergoing a paradigm shift. Traditional approaches, characterized by didactic lectures, static textbooks (1–3), and episodic clinical rotations, increasingly struggle to meet the demands of a rapidly evolving healthcare system. Today, medical students must acquire extensive biomedical knowledge while developing clinical reasoning, communication, empathy (2, 4), and procedural skills in settings constrained by time and patient safety. This growing complexity, together with the limits of conventional methods, has driven the search for more flexible, effective, and student-centered strategies (5).

Technological innovation has become a central driver of this transformation, with simulation platforms, e-learning modules, and digital assessment tools gaining widespread adoption (1, 4). Among these, artificial intelligence (AI) and extended reality (XR) stand out as particularly disruptive and promising, offering new possibilities for training future healthcare professionals (5, 6).

In medical education, AI underpins adaptive learning platforms, intelligent tutoring systems, virtual patient simulations, automated feedback mechanisms, and predictive analytics (7–10), enabling individualized learning trajectories and targeted instruction. XR, encompassing virtual reality (VR), augmented reality (AR), and mixed reality (MR), provides immersive learning experiences that simulate real-life medical scenarios (8–13). VR enables fully virtual environments (e.g., surgical procedures or emergency management), AR overlays digital information onto physical settings (14, 15), and MR integrates real and virtual elements, allowing interaction with patients or holographic devices in real time (16). XR is increasingly used to teach anatomy, diagnosis, surgical skills, and interprofessional collaboration in safe, controlled, and repeatable environments (17).

The integration of AI and XR into medical education has been accelerated by several converging forces (19). The rapid maturation and commercialization of these technologies have made them more accessible and affordable for academic institutions (17–19), The proliferation of consumer-grade VR headsets, cloud-based AI platforms, and open-source development tools has lowered implementation barriers (20, 21). The COVID-19 pandemic further catalyzed digital transformation in education (22): social distancing and the suspension of in-person clinical training compelled educators to adopt alternative modalities for delivering content and assessing competencies (23). AI- and XR-based solutions offered continuity alongside interactivity, scalability, and data-rich feedback (9, 21).

Evolving educational paradigms, including competency-based learning, flipped classrooms, and just-in-time training, align closely with the potential of smart and immersive technologies (24). Innovations that combine AI and XR can support real-time assessment of clinical decision-making, situated learning in realistic contexts, and the development of non-technical skills such as teamwork and communication (20, 25).

Growing evidence indicates that AI and XR can enhance student engagement, knowledge retention, and skill acquisition at various stages of medical training (26). VR simulations have improved procedural accuracy and confidence among trainees (19), while AI-based tutoring systems provide personalized feedback that may accelerate learning curves. XR applications in anatomical training enable dynamic, three-dimensional exploration beyond what static cadaver dissection (23, 27). However, high infrastructure costs, variable user acceptance, lack of faculty training, and institutional inertia hinder wider adoption (28). The use of AI raises ethical concerns around data privacy, algorithmic bias, and transparency, whereas XR platforms may induce motion sickness, demand substantial computational resources, and still fall short of the unpredictability of real-life clinical environments (29).

Existing research remains fragmented, often focusing on single technologies or small-scale implementations without evaluating long-term outcomes or cost-effectiveness (21, 25). There is a clear need for an integrated understanding of how AI and XR intersect, complement, and challenge each other within medical education (30).

This study synthesizes and critically analyzes the literature on the integration of AI and XR in medical education between 2019 and 2024, identifying gaps in knowledge and practice and outlining directions for future research and implementation. This narrative review adopts a multidimensional lens on the pedagogical, technological, ethical, and institutional aspects of AI and XR in medical education, focusing on how AI-only, XR-only, and emerging AI–XR interventions are currently used to support curriculum design, assessment, and preparation for clinical practice, rather than on head-to-head comparisons between the two modalities.

## 2 Methodology

This review adopted a structured and transparent methodological approach to identify, assess, and synthesize the existing literature on the integration of AI and XR technologies in medical education. The review process was based on the principles of systematic review methodology and elements adapted from the PRISMA (Preferred Reporting Items for Systematic Reviews and Meta-Analyses) framework, with the overall objective of retrieving empirical studies that report on the design, implementation, and evaluation of AI-, XR-, or combined AI–XR–based educational interventions in medical training settings (31, 32). Data collection focused exclusively on original research articles that provided measurable educational outcomes, such as knowledge acquisition, skill development, cognitive performance, or student engagement. The literature search and selection process was conducted in four sequential stages (database identification, initial screening, eligibility assessment, and final inclusion for intervention analysis). These phases relied on well-defined inclusion and exclusion criteria to ensure relevance, methodological quality, and alignment with the review objectives, and they served as the basis for a thematic and comparative analysis of trends, technologies, pedagogical strategies, and reported impacts.

### 2.1 Review design using PRISMA and PICO methodologies

To guide the methodological structure of this review, we used two widely recognized frameworks: PRISMA (Preferred Reporting Items for Systematic Reviews and Meta-Analyses) and PICO (Population, Intervention, Comparison, Outcome) (31). PRISMA was selected to promote clarity in literature selection and transparency in reporting (32), structuring the study identification and selection into four stages with specific steps at each phase.

The PICO methodology was chosen because it allows for the formulation of structured inclusion criteria and helps maintain thematic consistency among selected studies (33). It is particularly useful for identifying articles focused on interventions that align with the scope of the review. This PICO framing, including the research question, was defined *a priori* before data extraction, and guided both the construction of the search string and the subsequent screening process. The population considered included learners across the continuum of medical and health-professions education, namely undergraduate medical students, residents, and fellows in graduate medical education, and practicing clinicians engaged in continuing professional development. No limits were imposed on clinical specialty, provided that participants were enrolled in a structured educational or training activity.

Interventions were grouped into three categories: (i) AI-only educational applications (e.g., natural language processing tools, generative models, intelligent tutoring systems); (ii) XR-only applications (virtual, augmented, or mixed reality–based simulations); and (iii) integrated AI–XR interventions in which both components were used within the same educational activity. Comparators included traditional teaching methods and non-intervention conditions, and outcomes focused on learning effectiveness. Four outcome domains were defined *a priori*: knowledge (scores on written or structured theoretical tests), skills (performance on practical or procedural tasks), performance (objective indicators such as error rates, completion time, or simulator-derived scores), and engagement (self-reported or behavioural indicators of motivation and participation). The main assessment instruments for each study are summarized in Table 5, and the overall PICO framing in Table 1.

**Table 1:** PICO framework used to define eligibility criteria for included studies.

	Element	Description
P	Population	Medical students, healthcare trainees, and educators
I	Intervention	Educational use of AI, XR, or combined AI–XR technologies (e.g., NLP, generative models, immersive VR/AR/MR simulations)
C	Comparison	Traditional instruction or no intervention
O	Outcome	Learning outcomes related to knowledge acquisition, skill development, task performance, and learner engagement.

The model guided the identification of relevant literature focused on AI-, XR-, and combined AI–XR–based educational interventions and their impact on learning outcomes in medical and healthcare training contexts.

Using the PICO model, the research question was iteratively refined to align with the objectives of this review: to explore the educational impact of Artificial Intelligence and Extended Reality technologies in medical and healthcare training. The final research question guiding this review was:

“*In empirical studies published between 2019 and 2024, how have AI-only, XR-only, and combined AI–XR technologies been used in medical and health-professions training, what learning outcomes have been reported, and what implementation challenges have been described, with specific attention to the limited number of fully integrated AI–XR interventions?”*

Given the limited number of fully integrated AI–XR studies, we also retained AI-only and XR-only interventions to contextualize the emerging evidence base and identify design patterns for future AI–XR ecosystem development. Guided by our research question and the PICO framework, we implemented a structured Boolean search organized into three concept clusters combined with AND—(1) AI-related terms (e.g., artificial intelligence, natural language processing, generative AI), (2) medical and health-professions education and training, and (3) instructional modality (e-learning, simulation, virtual reality)—with synonyms within each cluster combined with OR. The complete, database-specific search strings are summarized in Table 2.

**Table 2:** Database search results using structured boolean queries focused on the intersection of AI and medical education, in the range 2019-2024.

Database	Query	Results
Scopus	"Artificial Intelligence” OR “Natural Language Processing” OR “Generative AI” AND “Medical Education” OR “Healthcare Training” AND “E-learning” OR “Simulation-based learning” OR “Virtual Reality"	258
PubMed		84
IEEE Xplore		50

### 2.2 Database selection and search strategy

To ensure a comprehensive and multidisciplinary collection of articles, three major academic databases were selected for this review: SCOPUS, PubMed, and IEEE Xplore. Each database contributes uniquely to the intersection of healthcare, education, and emerging technologies, making them essential for capturing diverse research perspectives.

SCOPUS was selected for its extensive indexing of peer-reviewed journals in health sciences, education, and engineering.
PubMed was selected for its rigorous selection of biomedical literature, including clinical and educational research specific to healthcare training.
IEEE Xplore was included to capture technical and engineering contributions to AI and simulation technologies, particularly those not indexed in traditional medical databases.

The Boolean formulation of main, topic-centric keywords (Table 2) was designed to encompass research at the intersection of AI technologies and immersive educational methods in healthcare, including simulation and virtual reality as core XR modalities. The study period was limited to 2019–2024, a timeframe characterized by exponential advances in generative AI relevant to adaptive learning and intelligent tutoring systems, as well as the digital transformation catalyzed by the COVID-19 pandemic (2020-2022). This restriction was intended to capture the most up-to-date and relevant literature, including both early adoption studies and recent developments in the field.

The search strategy employed in these databases yielded a diverse set of studies spanning the technological, educational, and clinical dimensions of AI in medical education. Boolean queries were applied uniformly, and the results reflect the multidisciplinary scope of the field. Table 2 presents a concise summary of the query structure and the number of articles retrieved from each source, providing a fundamental dataset for the screening and eligibility stages detailed in the following sections.

**Figure 1:**
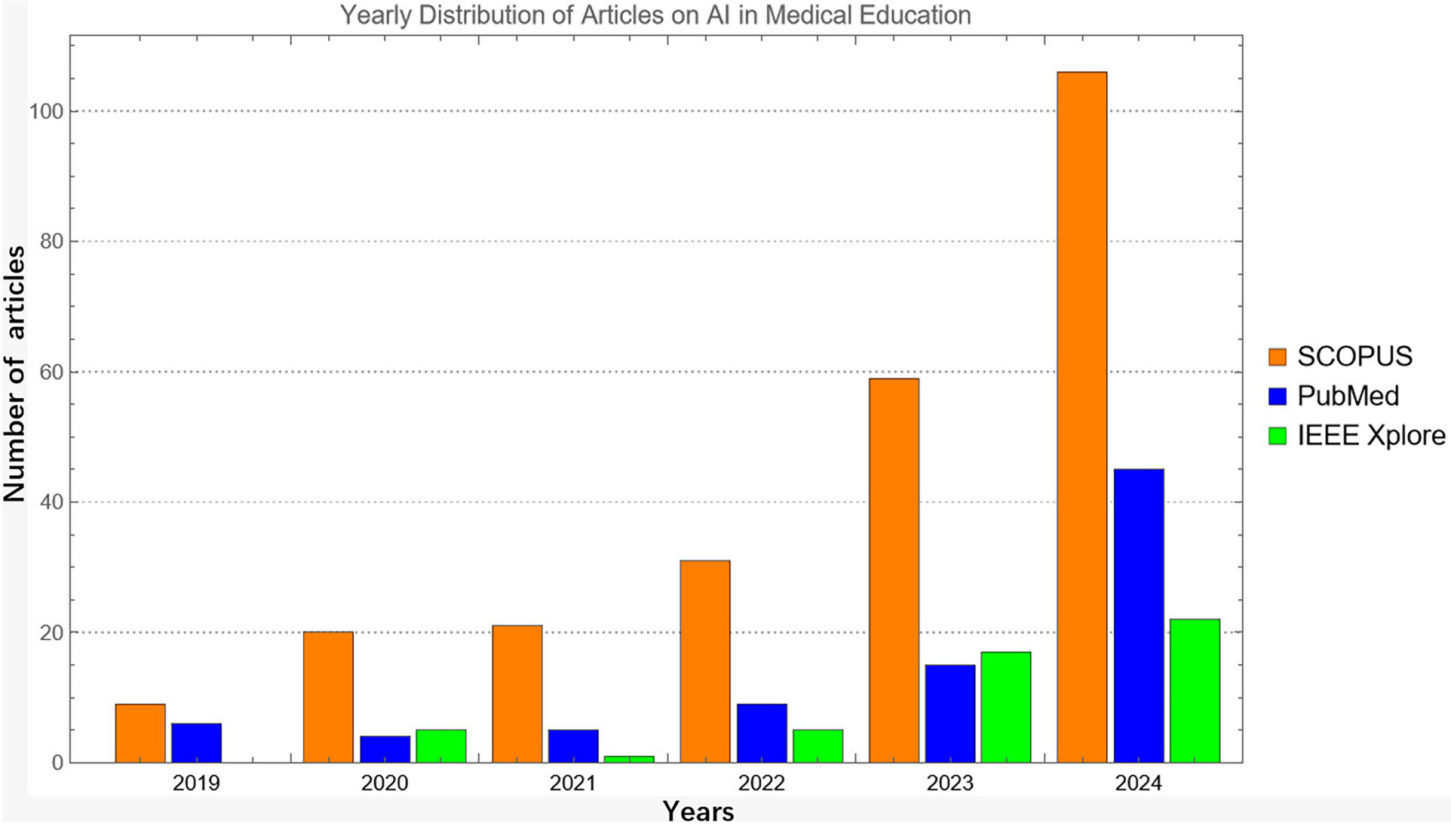
Annual distribution of all records retrieved in scopus, pubMed, and IEEE xplore between 2019 and 2024 prior to screening, eligibility assessment, and final inclusion.

The volume and temporal evolution of these retrieved records provide insight into how interest in AI within medical education has developed over time. As shown in Figure 1, there is a clear increase in the number of records identified in all three databases from 2020 onwards, with SCOPUS consistently reporting the highest annual counts, followed by PubMed and IEEE Xplore. This trend reflects broader growth in research on AI and immersive digital tools in medical education rather than the characteristics of the final 13 studies included in the review.

### 2.3 Identification

The identification phase involved a systematic search of the three main databases mentioned above: SCOPUS (*n* = 258), PubMed (*n* = 84), and IEEE Xplore (*n* = 50). The search focused on literature specifically examining how AI technologies are integrated into teaching, learning, and assessment processes within medical education.

**Figure 2:**
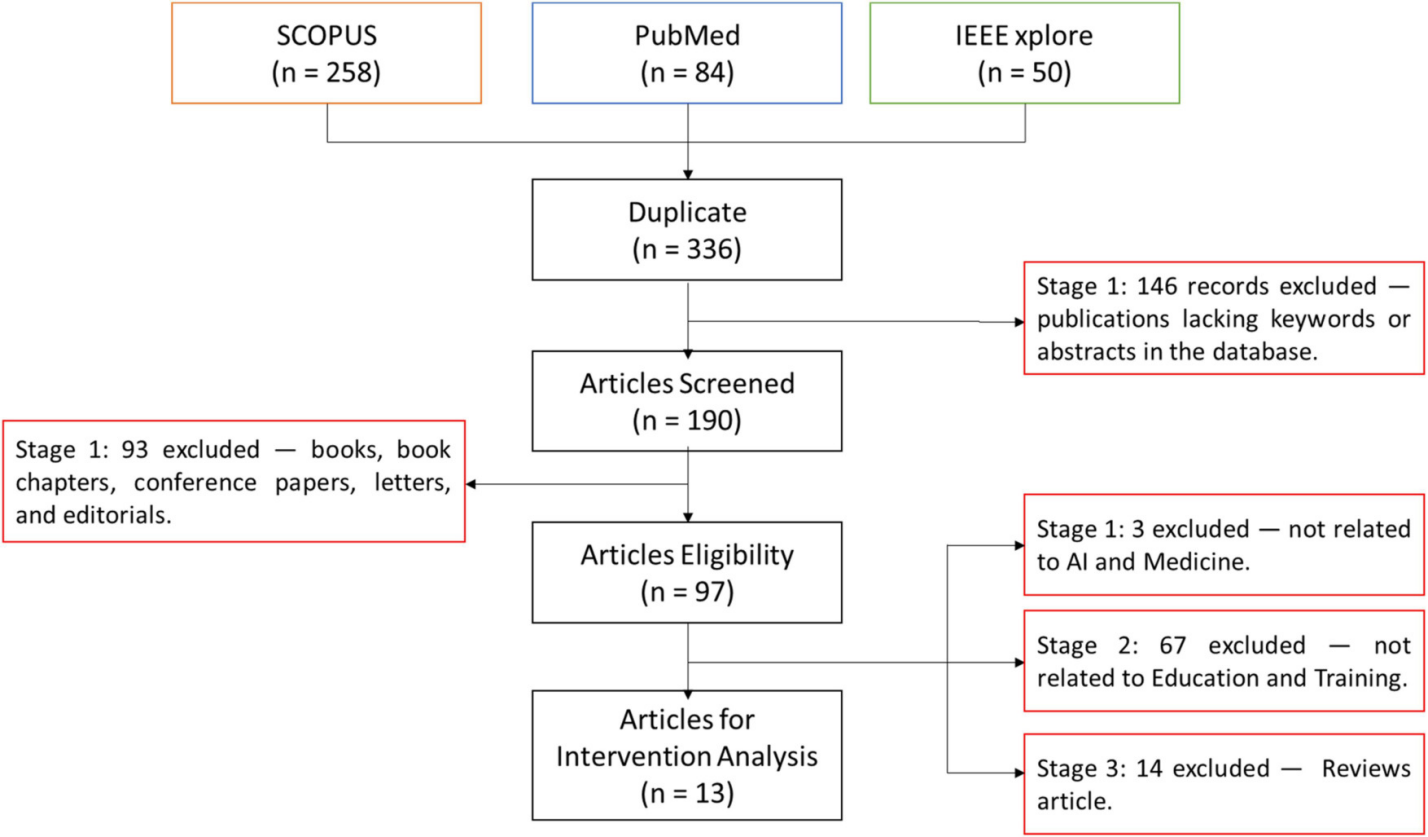
PRISMA flowchart showing the selection process of studies on AI in medical education across four phases: identification, screening, eligibility, and inclusion.

Figure 2 shows how the initial search yielded 392 records. The search strategy was intentionally designed to be highly sensitive, in line with PRISMA-oriented guidance, to minimize the risk of missing empirical AI- and XR-based educational interventions. Consequently, many records identified at the identification stage were concept papers, narrative or systematic reviews, or clinical AI studies without an educational component, which were subsequently excluded during the eligibility assessment.

After removing duplicates, 336 records remained. During title/abstract screening, records lacking essential metadata (e.g., abstracts or keywords) were excluded (*n* = 146), leaving 190 records for full-text review. At full-text screening, non-peer-reviewed formats (books, book chapters, conference papers, letters, and editorials) were excluded (*n* = 93), yielding 97 eligible articles. Finally, we excluded 3 studies not related to AI and Medicine, 67 not related to Education/Training, and 14 review articles, resulting in 13 studies included in the intervention analysis.

### 2.4 Screened

After removing duplicates, 336 records were available for the initial assessment. The goal of the screening phase (title/abstract screening) was to retain only records clearly relevant to the application of AI in medical and health-professions education for full-text eligibility. Each record was checked for title, abstract, indexed keywords, and basic bibliographic completeness.

Records lacking essential metadata needed to judge relevance—principally abstracts and/or indexed keywords (a frequent issue in technical databases such as IEEE Xplore)—were excluded.

All exclusions were logged to ensure transparency and reproducibility. In total, 146 records were excluded at this stage, leaving 190 records for full-text eligibility assessment. The outcomes of the screening step are summarized in Figure 2.

### 2.5 Eligibility

The eligibility process involved the exclusion of 93 records based on publication type and source format. The excluded articles included books, book chapters, conference proceedings, editorials, letters, and commentaries, which, during the full-text review, were identified as not contributing information to the inclusion criteria. Recognizing that some of these sources provided important conceptual descriptions or theoretical perspectives on AI and its potential applications in medical education, they were rejected due to their preliminary nature, which often limits methodological detail and omits follow-up results or student assessment data.

In the case of editorials and letters, while sometimes relevant to ongoing debates in the field, they were also excluded due to their opinion-based structure and lack of data-driven findings. Books and book chapters, although often comprehensive, present challenges related to accessibility, inconsistent peer review standards, and variability in how interventions and outcomes are reported.

The exclusion of these 93 articles was necessary to preserve the analytical integrity and focus of the review, maintaining the focus on information extraction specifically in original articles. Therefore, only full-text empirical studies were eligible for further analysis. After this filtering process, 97 full-text articles were retained, which constitute the eligibility stage, where their relevance to AI applications in medical education and the presence of measurable learning outcomes were further assessed.

### 2.6 Inclusion

Of the 97 full-text articles assessed for eligibility, 13 studies were included in the final analysis. The selection of this resulting number of studies for inclusion was the result of a rigorous three-stage selection process, designed to isolate only those studies that met all thematic, methodological, and empirical criteria related to the central focus of the review: the use of AI in medical education.

The first stage of exclusion focused on articles unrelated to AI and medicine, of which 3 were identified, leaving 94. The eliminated studies were related to AI in a general or metaphorical sense, but did not involve actual AI systems, models, or algorithms in medical education contexts and focused on training in disciplines not directly related to medicine.

In Stage 2 of the eligibility assessment, we excluded technical and clinical journal articles that addressed the development or validation of AI systems for diagnosis, risk prediction, or clinical workflow optimization, but that did not involve students or evaluate educational interventions and therefore did not align with the review objective of synthesizing evidence on AI-based teaching and learning practices. These records were coded as having no AI-based educational applications because they did not report implemented AI, XR, or combined AI–XR tools used in teaching, learning, assessment, or simulation for health-professions learner. Thirty-four articles were excluded at this stage, leaving 67 studies for the final filter.

To maintain the focus on original and empirical research, review articles, both narrative and systematic, were then excluded from the dataset. Systematic, scoping, and narrative reviews were therefore treated as non-eligible study designs and were used only for contextual background and to cross-check whether our search had missed primary studies (*n* = 14 excluded). While informative, these articles summarized existing literature rather than presenting new data or intervention results. The exclusion of these studies means that the final synthesis is based solely on primary studies with defined populations, interventions, and measurable learning outcomes.

The 13 articles ultimately selected constitute the inclusion stage. These are peer-reviewed, intervention-based research on the applications of AI in medical education, which included a quantitative or qualitative assessment of the educational impact.

In terms of study design, the presence of a control group was not used as an inclusion criterion. Many AI- and XR-based educational interventions are still reported as pilot or feasibility work and therefore lack formal comparators. During data extraction, the presence and type of comparator or control condition were recorded for each study and, when applicable, classified into one of four categories: (i) no formal comparator (single-group or pre–post design), (ii) traditional instruction or standard curriculum, (iii) alternative technology-enhanced instruction without AI or immersive XR, and (iv) historical or convenience cohorts.

### 2.7 Quality appraisal

All included studies underwent a structured quality appraisal to assess the robustness of their design and reporting. Each article was evaluated using design-appropriate critical appraisal checklists (e.g., tools for randomized and quasi-experimental studies, observational designs, and qualitative research). When assessments differed, they were reconciled through discussion until consensus was reached. The appraisal focused on key domains such as study design and setting, sampling strategy and sample size justification, clarity and validity of outcome measures, handling of confounding factors, and transparency of data analysis and reporting. Given the heterogeneity of study designs and outcomes, we did not compute a pooled quality score. Instead, studies were categorized qualitatively as offering lower, moderate, or higher methodological robustness, and these judgements informed the synthesis presented in the “Methodological Considerations and Quality of Evidence” section.

## 3 Results

The 13 selected studies detail how AI and XR have been implemented in medical and health-professions education, focusing on their effects on learning outcomes, implementation limitations, faculty preparedness, and ethical considerations.

Of these studies, only a small subset explicitly combined AI components with XR environments within a single educational intervention. Most articles evaluated either AI-based tools (e.g., generative AI, machine-learning–driven analytics, intelligent tutoring) or XR-based simulations without an AI layer. The synthesis therefore distinguishes, where possible, between AI-only, XR-only, and emerging integrated AI–XR designs.

### 3.1 Analysis of the educational impact of the use of AI and XR in medicine

Table 3 presents an overview of the studies, their variables, methodologies, and sample sizes. Five studies focused on engagement, defined as the emotional and cognitive involvement of learners in learning activities. These interventions often used immersive or personalized environments to increase motivation and attention (34) surveyed 406 healthcare professionals and found widespread enthusiasm for XR-based training environments. However, the cross-sectional design limited causal inference. Similarly (38), explored how AI and VR were perceived in virtual interviews, with results indicating adaptability and a positive reception among 112 participants. In the study by (45), they demonstrated the reach of an international e-learning platform with NLP/ML support, with over 1,600 participants. Despite the large sample size, the study acknowledged limitations in assessing long-term impact (46). analyzed radiology training during the COVID-19 pandemic, highlighting the role of virtual platforms in maintaining student engagement (36), while focusing on communication skills, reported that AI-generated case simulations increased student participation due to their interactivity and realism.

**Table 3:** Summary of the studies and their intervention, educational variable evaluated, technology used, stage of development and sample size.

Study number	Intervention	Variable	Limitations	Stage of employed technology	Technology type	Number of participants
Khan et al. (34)	Survey of healthcare professionals’ perceptions of XR adoption	Engagement	Cross-sectional design limits generalizability	Study	Not reported	406
Bonfitto et al. (35)	ChatGPT dialogues simulate radiographer–patient interactions for claustrophobia	Skill development	Small pilot, ChatGPT not designed for simulation	Pilot	ChatGPT (GPT-3.5 & GPT-4)	6
Artemiou et al. (36)	AI-generated cases & standardized clients for veterinary communication training	Skill development	Single institution, limited familiarity	Study	ChatGPT-3.5	237
Prevezanou et al. (37)	ML models classify progress on laparoscopic VR simulator tasks	Performance	Limited kinematic data and subjective labels	Development	Machine learning algorithms	23
Tolentino et al. (38)	Survey on virtual interviews and AI/VR integration in residency programs	Engagement	Single-institution cross-sectional design	Study	Not reported	112
Latour et al. (39)	VASN features aid FESS simulation training for otolaryngology trainees	Performance	Small sample & simulation environment	Study	Virtually Augmented Surgical Navigation	15
Real et al. (40)	VR curriculum with didactics & simulations for pediatric residents	Skill development	Small sample & single institution	Pilot	Virtual Reality simulation	55
Krive et al. (41)	Four-week AI course integrating evidence-based medicine & clinical topics	Skill development	Small cohorts & single institution	Study	General AI instruction	20
Mergen et al. (42)	Development of AI-driven VR platform for virtual patients (medical tr.AI.ning)	Skill development	Development stage—no evaluation	Development	AI-driven VR platform	Not reported
Tsopra et al. (43)	Elective program where students design AI clinical decision support systems	Skill development	Small elective & limited generalizability	Study	AI-CDSS design	15
Andersen et al. (44)	Interactive online platform (VIOLA) for diabetic retinopathy training	Skill development	Limited to regional adoption	Study	Online learning platform	150
Borakati et al. (45)	International e-learning course evaluated with NLP & ML	Engagement	Limited generalizability & outcome measures	Study	E-learning with NLP/ML	1611
Gabr et al. (46)	Impact analysis of radiology case volumes & remote education during pandemics	Engagement	Observational and single-institution data	Study	Virtual learning platforms	Not reported

**Figure 3:**
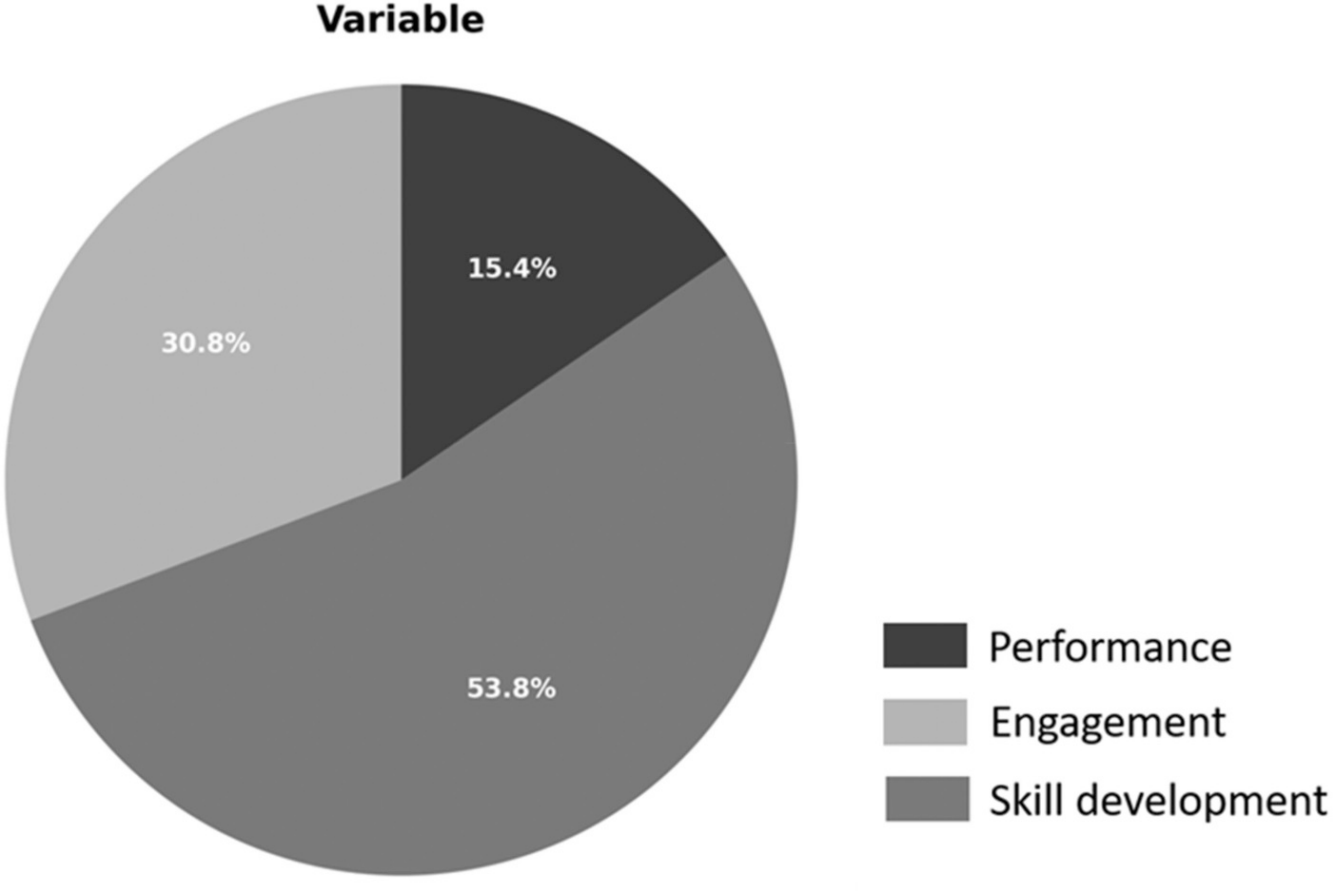
Distribution of the reviewed studies according to the main educational variable: skills development, engagement and performance.

Figure 3 details that engagement-oriented studies represented 38% of the sample. These findings suggest that AI and XR can effectively foster student participation, especially when traditional formats are disrupted or inaccessible. Skills development emerged as the most frequently assessed educational variable, appearing in seven of the 13 studies (54%) (35). conducted a pilot study on the use of ChatGPT to simulate conversations between radiology technicians and claustrophobic patients. Although the sample size was limited (*n* = 6), the study demonstrated the potential for developing communication skills through AI-generated dialogue (36). These studies used standardized AI-generated cases in veterinary training (*n* = 237) and reported improvements in confidence and fluency during client interactions.

Other studies, such as (40), employed a virtual reality simulation program for pediatric residents, combining theory with practice. In this study, participants showed improved task performance, although the institutional scope was limited. In another application (41), offered a four-week AI course that integrated clinical and evidence-based medicine content, fostering critical thinking despite small group sizes (43). Their research encouraged students to design their own clinical decision support systems, integrating AI tools into the curriculum design: a promising approach to skills development through hands-on experience (44). They developed the VIOLA platform for diagnosing diabetic retinopathy, training 150 participants via an online interface. Finally (42), described an AI-powered VR platform, currently under development, and proposed future training in virtual patient encounters.

**Figure 4:**
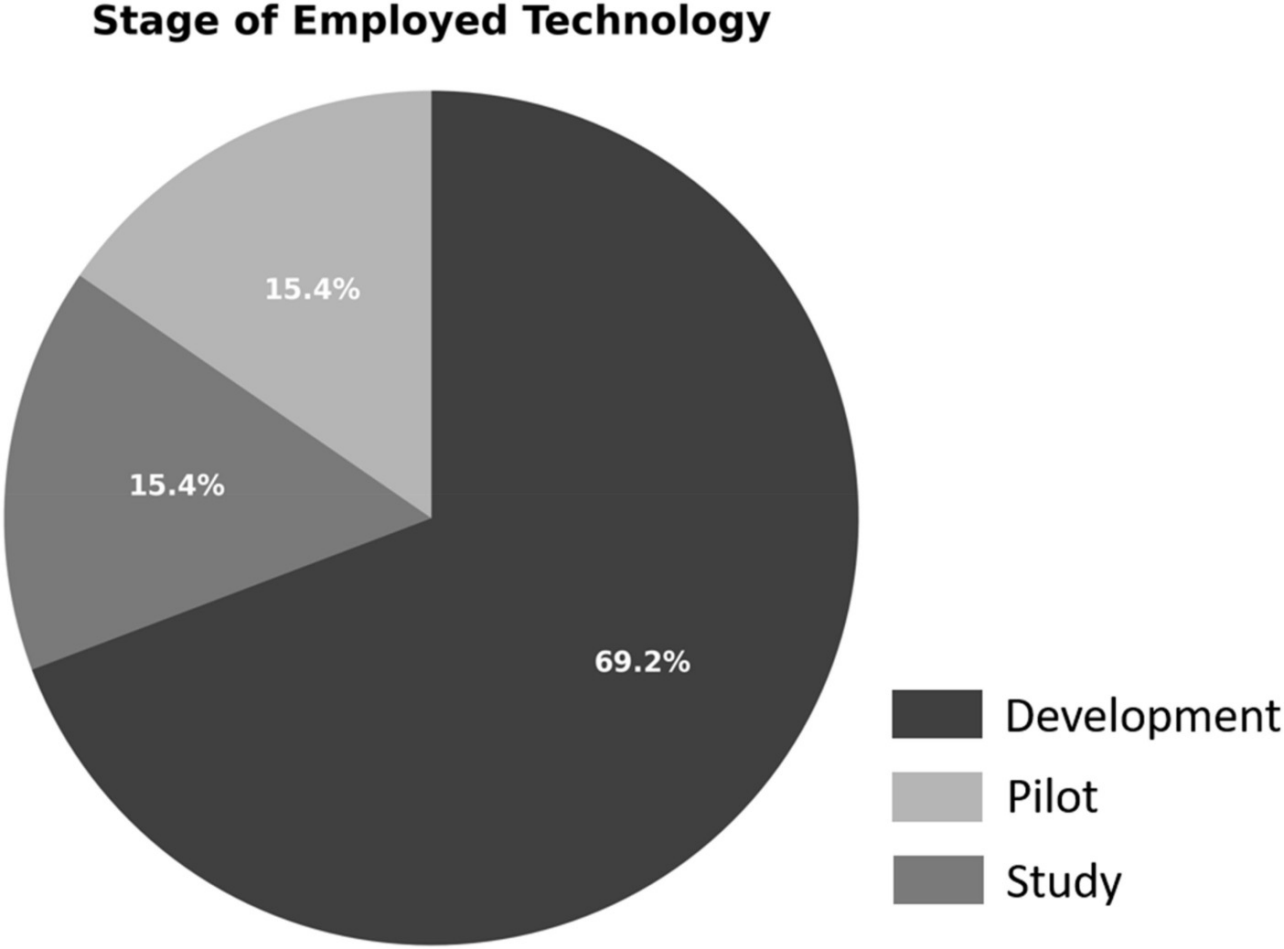
Studies reviewed by technology implementation stage: study, pilot, and development phases.

Only two studies (15%) explicitly measured performance, defined as the demonstration of competence in clinical tasks or simulations (37). used machine learning models to classify students' progress in laparoscopic virtual reality simulations. Despite the innovative analysis, the small sample size (*n* = 23) and reliance on subjective classification limited the robustness of the conclusions (39). tested Virtual Augmented Surgical Navigation (VASN) in otolaryngology simulation. The intervention improved performance in surgical planning but was limited by the sample size (*n* = 15) and simulation fidelity. As shown in Figure 4, performance remains the least explored outcome in the current AI/XR education literature.

Two studies were identified as pilot initiatives, representing initial experiments to explore feasibility and usability (35). conducted a pilot study on the use of ChatGPT for radiologist and patient simulations, and (40) implemented a VR pilot program; two other studies were in the development stage, where no outcome assessment had been performed (42). proposed an AI-powered VR platform but did not present student data. The existence of such nascent projects highlights the innovative nature of this research area but also points to the need for more rigorous validation frameworks.

The distribution shown in Figure 4 reflects a steady flow of innovation, progressing gradually from exploratory design to empirical validation. However, the relatively low number of pilot and development projects could imply a publication bias toward studies with proven technologies, which might overlook the value of documenting conceptual innovations or prototypes.

### 3.2 Observed effects, challenges, and methodological contexts

In general, studies consistently documented improvements in educational performance, student confidence, and skill acquisition (34). conducted a cross-sectional survey in Pakistan to assess perceptions and readiness for integrating XR into medical education. The results indicated that 83.8% of participants believed XR could effectively improve the quality of education and patient care, observing statistically significant correlations between familiarity with XR and favorable attitudes toward its use in diagnostic and surgical procedures.

Table 4 summarizes the observed intervention effects together with study design and comparator. In this review, effects were classified as “positive” when the original study reported improvement in at least one predefined learning outcome (knowledge acquisition, skill development, performance, or engagement), either relative to baseline in pre–post designs or compared with a control or alternative condition, using the significance criteria specified by the authors. Outcomes were labeled as “mixed/neutral” when no clear improvement was observed or when findings differed across domains.

**Table 4:** Summary of included studies: study design, comparator/control condition, and observed intervention effects across predefined learning outcomes.

Study number	Effect	Barriers/challenges	Study design	Comparator/control condition
Khan et al. (34)	Positive	Technological, privacy and training barriers	Cross-sectional survey	None (no comparator; single-group cross-sectional survey)
Bonfitto et al. (35)	Positive	AI bias and model differences	Pilot case study	None (single-group pilot; no educational control group)
Artemiou et al. (36)	Positive	Data privacy, unclear guidelines, low literacy	Descriptive study	None (single-group communication lab; no comparator group)
Prevezanou et al. (37)	Positive	Restricted data access and sensors	Developmental study	None (single-group VR training curriculum; no external comparator)
Tolentino et al. (38)	Positive	Response bias and limited generalizability	Cross-sectional survey	None (no comparator; observational survey of AI education initiatives)
Latour et al. (39)	Positive	Technology complexity	Prospective trial	Within-subject repeated-measures design (participants serve as their own control across VASN sessions)
Real et al. (40)	Positive	Cost and resource limitations	Randomized controlled pilot trial	Active control group—alternative VR simulation (respiratory-distress scenario; intervention vs. control VR curriculum)
Krive et al. (41)	Positive	Faculty expertise and funding limitations	Educational innovation	None (single-group curriculum implementation; no separate control group)
Mergen et al. (42)	Increased	Technical complexity and integration	Development project	Not applicable (development project; no learner cohort or control group)
Tsopra et al. (43)	Positive	Logistical challenges	Elective course evaluation	None (single-group elective course evaluation; no comparator group)
Andersen et al. (44)	Positive	Need robust digital infrastructure	Case study/implementation	None (single-group implementation; no comparator group)
Borakati et al. (45)	Positive	Digital infrastructure & language barriers	Cross-sectional evaluation	None (single-group international e-learning evaluation; no comparator group)
Gabr et al. (46)	Increased	Reduced case volumes & remote challenges	Observational analysis	Historical control—comparison with pre-pandemic case volumes from prior years

Regarding study designs and comparators, we observed a predominance of single-group or pre–post evaluations. Only a minority of studies implemented a formal control group, typically based on traditional lectures or standard skills training, while a smaller subset compared AI or XR interventions against other digital resources or simulation modalities. The type of comparator used in each study is summarized in the “Comparator/control condition” of Table 4.

Similarly (40), in a randomized controlled pilot trial, demonstrated that residents participating in a VR-based behavioral health training module showed statistically significant improvements in motivational interviewing (MI) and anticipatory behavioral health guidance (OAHG) skills. These improvements were quantitatively supported by a higher frequency of open-ended questions and MI-aligned behaviors, illustrating the effectiveness of immersive simulation as a teaching tool.

Another noteworthy contribution comes from (35), who used ChatGPT-3.5 and ChatGPT-4 to simulate interactions with patients in a radiographic environment. This pilot case study showed a 96.7% success rate in managing virtual patients experiencing claustrophobia during magnetic resonance imaging (MRI), with communication styles adapted to the different experience levels of the radiologic technologists. This innovative application of LLM models adds a new dimension to preclinical training by reinforcing communication skills between students and patients.

**Figure 5:**
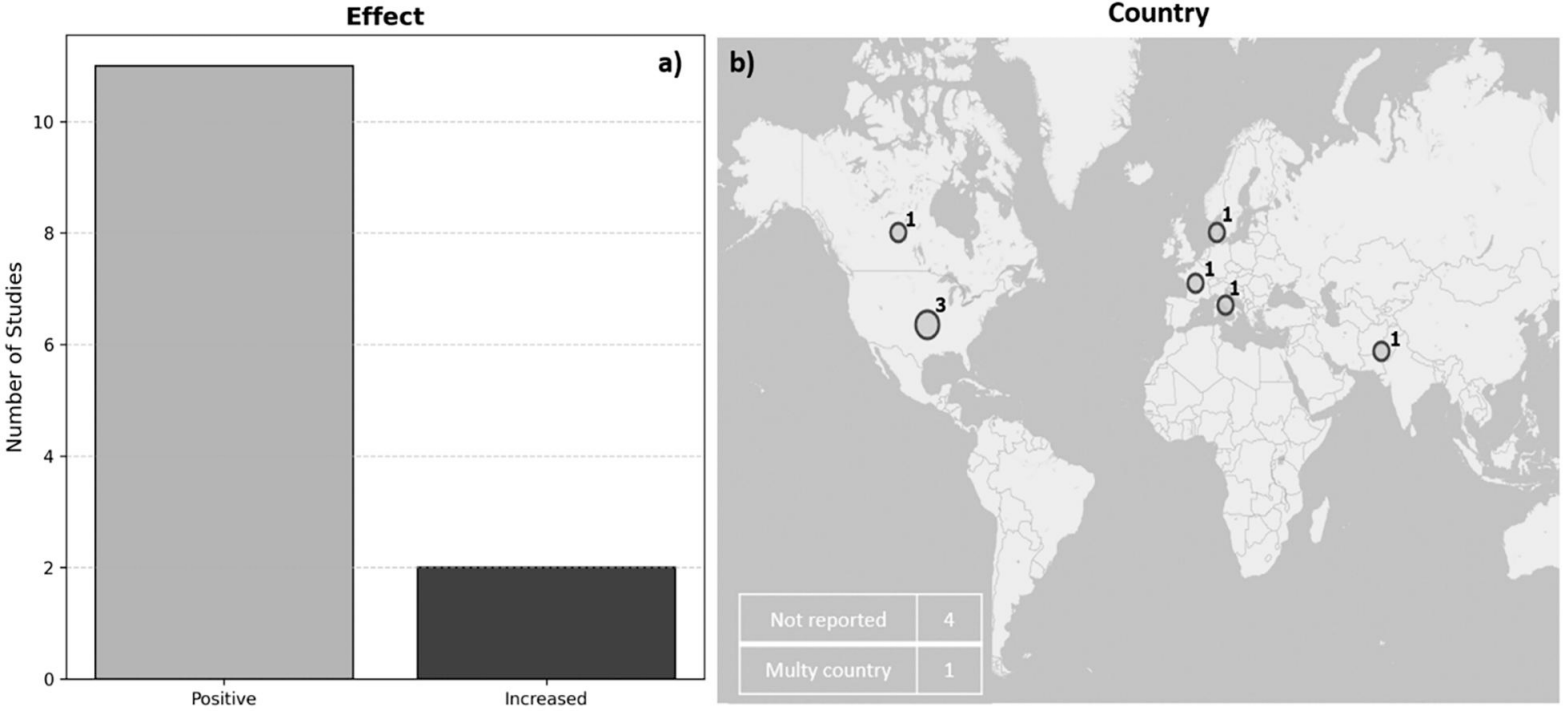
**(a)** distribution of the educational effects reported in the selected studies. **(b)** Geographic distribution of the studies by country, including multiple locations and cases with unspecified origin.

Figure 5a shows that 11 of the 13 studies (84.6%) reported a positive impact on learning or teaching delivery, whereas 2 (15.4%) were mixed/neutral. Figure 5b shows the varied geographical distribution of the studies, with contributions from North America (*n* = 4), Europe (*n* = 4), Asia (*n* = 1), and one multicenter study. Four studies did not specify their location, which limited a more detailed geographical analysis. The United States had the largest representation (*n* = 3), with studies focusing on innovative applications such as digital radiology training during COVID-19 (46) and competency-based AI training (41). The multicenter evaluation in (45) revealed the scalability of e-learning platforms and explored the use of natural language processing to analyze user feedback in 24 countries, providing insights into the global applicability of AI-enhanced learning.

Despite the positive results in the vast majority of studies, almost all reported significant barriers hindering smooth adoption. Table 4 shows technological complexity and system integration problems detailed in (39) and (42), as well as concerns about data privacy and a lack of regulatory clarity observed in studies (34) and (36). Another key barrier, according to (41) and (45), is inadequate infrastructure and training for carrying out study activities, compounded, as in the case of (40), by high costs and resource limitations.

A recurring theme in various studies is the ethical and legal ambiguity associated with the use of AI and XR in educational settings. Study (34) identified privacy concerns as one of the main barriers (mentioned by 63.9% of participants), along with technological underdevelopment (70%) and insufficient XR training among healthcare professionals (45.8%) (36)., detailing a centralization in the use of AI in veterinary education, observed that upper-level students showed resistance due to uncertainty and a lack of confidence in AI tools, pointing to a systemic need for early training in AI digital literacy. Similarly (43),, through their elective AI-CDSS course implemented in France, reported logistical barriers to implementation, although student participation remained high.

### 3.3 Areas of medical education, training modalities, and implementation environments

**Figure 6:**
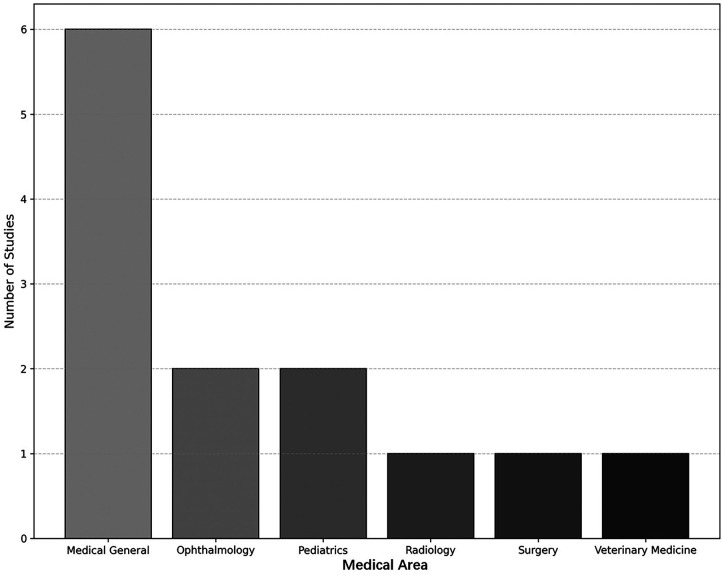
Distribution of studies by area of medical education.

Table 5 shows that current evidence reveals a dynamic integration of AI and immersive technologies across a wide range of medical education settings; these innovations offer promising strategies for skills acquisition, communication training, and clinical reasoning. As shown in Figure 6, general medical education represented the most common area of ​​focus, encompassing almost half of the reviewed studies, a total of 6. This finding reflects a growing recognition of the critical role that digital health literacy plays in modern clinical practice. Generalist programs, such as those implemented by (34) (41),, and (43), frequently addressed key concepts of AI, clinical decision support systems (CDSS), telemedicine, and data ethics. On the other hand, the study by (41) presented a four-week interactive course that used clinical practice simulations and online modules to teach medical students the application of AI in patient care. This course demonstrated a high level of participation and knowledge acquisition, underscoring the importance of integrating AI literacy from the early stages of medical curricula.

**Table 5:** Area of medical education, duration of the intervention, assessment tools, training modalities and implementation contexts.

Study number	Area	Duration of intervention	Assessment tools	Training provided	Context/setting
Khan et al. (34)	Medical General	Two-month survey	Online questionnaire	None	Healthcare community across Pakistan
Bonfitto et al. (35)	Radiology	Not reported	Simulation success and error rates	Not reported	Radiography education (MRI)
Artemiou et al. (36)	Veterinary Medicine	90-minute lab	Questionnaire and perceptions survey	30-min theory+60-min practice	Veterinary school communication lab
Prevezanou et al. (37)	Surgery	Not reported	VR task metrics & ML classification	Structured VR curriculum	Laparoscopic simulation lab
Tolentino et al. (38)	Medical General	Not reported	Online survey	None	Residency program at McGill University
Latour et al. (39)	Surgery	Not reported	Technical skill scores and time	VASN simulation sessions	3D-printed FESS simulation lab
Real et al. (40)	Pediatrics	Four months	Psychologist-rated VR scenarios	Didactics+VR practice	Pediatric residency training
Krive et al. (41)	Medical General	Four weeks	Quizzes and assignments	Interactive modules and assignments	University of Illinois College of Medicine
Mergen et al. (42)	Medical General	Not applicable	Not applicable	Not applicable	Not applicable
Tsopra et al. (43)	Medical General	Not reported	Student ratings and feedback	Lectures & brainstorming sessions	University Paris Cité
Andersen et al. (44)	Ophthalmology	Self-paced	Exercises & certification tests	Instructional videos & interactive tasks	Diabetic retinopathy screening training
Borakati et al. (45)	Medical General	Not reported	Sentiment analysis & topic modelling	E-learning modules	International multi-country
Gabr et al. (46)	Radiology	April 2020	Case volume & didactic hours analysis	Increased remote didactics	Radiology residency program

Surgery was explored in two studies (37, 39). In the first study, a VR-based structured laparoscopic curriculum was evaluated using machine learning classifiers to assess student performance. The incorporation of explainable AI (XAI) models into the feedback process allowed for objective, real-time performance monitoring (39). In their study, augmented surgical navigation (VASN) was used to enhance training in functional endoscopic sinus surgery (FESS). Their results detail that participants demonstrated significant improvements in their skill and confidence scores, supporting the effectiveness of AI-assisted simulation environments for surgical training.

Radiology was the focus of two additional studies (35). In their study, generative AI (ChatGPT-3.5 and 4.0) was used to simulate communication between radiology technicians and claustrophobic patients. The study highlighted the usefulness of AI in developing interpersonal skills through natural language interaction, an area often overlooked in traditional radiology training. In the same vein (46), researchers analyzed the impact of COVID-19 on radiology case volume and instructional design, observing a shift toward online remote learning platforms. These studies underscore how AI can facilitate both the technical and interpersonal aspects of radiology training.

Other fields included pediatrics (40), ophthalmology (44), and veterinary medicine (36). For example (40), a randomized controlled trial was conducted that incorporated virtual reality simulations to improve behavioral health anticipatory guidance (BHAG) and motivational interviewing skills among pediatric residents. The results indicated statistically significant improvements in communication skills, illustrating the potential of immersive technologies in behavioral pediatrics. In ophthalmology (44), the study developed a self-learning digital platform for diabetic retinopathy detection. The program offered certification and interactive learning tasks, and more than 150 healthcare professionals completed the course, demonstrating the scalability of this type of model. In contrast, research by (36) presented veterinary students with AI-generated standardized clients, providing valuable information on integrating AI into veterinary communication training. The students responded positively but identified a clear need to increase AI literacy within veterinary education.

The studies analyzed employed diverse training modalities, reflecting both institutional resources and pedagogical preferences. These modalities included self-paced online modules (44), structured simulation labs (39), short workshops (36), randomized controlled trials (40), and e-learning courses with natural language processing (NLP)-based assessments (45). For example (34, 38), were survey-based and did not involve direct educational interventions, but rather assessed perceptions and attitudes toward AI and XR in medical training settings.

The duration of the educational interventions varied significantly, from single-day or single-session labs (36) to modules lasting several weeks (41). In study (40), monthly instructional sessions were conducted over a four-month period, combining theory with repeated virtual reality practice. In comparison with studies (35, 37, 46), these studies did not report the duration of the intervention, highlighting the need for standardized reporting practices in medical education research.

Assessment tools ranged from validated questionnaires and psychometric scales to advanced analytical frameworks, including machine learning. Study (35) evaluated simulation success rates and AI error detection, while study (37) used predictive models to assess skill progression in laparoscopic tasks. Study (41) relied on weekly questionnaires and task ratings to monitor student learning outcomes, and study (40) employed pediatric psychologists to code residents' behavior in simulated encounters. Qualitative feedback was also common (43). incorporated student self-reports and project engagement metrics to measure curriculum effectiveness, while (45) applied sentiment analysis and theme modeling to extract qualitative feedback from large-scale courses.

Across the 13 studies, knowledge acquisition was typically measured through written tests or online quizzes; skill development and performance were captured through OSCE-style checklists, simulator-derived scores, error rates, and task-completion times; and engagement was assessed using Likert-type satisfaction or engagement scales, usage statistics from digital platforms, and qualitative feedback. This mapping between outcome domains and assessment instruments is summarized in Table 5.

Finally, some studies, such as (42), described digital education platforms under development, including “medical tr.AI.ning,” a modular tool designed to simulate first-person clinical decision-making using AI-powered virtual agents. These projects point to the future of medical education: scalable, customizable, and highly interactive environments powered by AI and XR.

## 4 Discussion

Most of the included studies reported positive effects associated with the implementation of AI and XR, particularly in improving skills development, student engagement, and performance indicators. These technologies were frequently introduced through simulations, virtual scenarios, chat-based AI, or e-learning platforms, suggesting a wide range of implementation modalities adapted to institutional resources and educational objectives. General medical education emerged as the most frequently addressed area, possibly reflecting its broad relevance and lower technical barriers to implementation compared to highly specialized fields. The widespread adoption of these innovations is not without challenges. Several of the selected studies highlighted recurring barriers, such as limitations in technological infrastructure, concerns about data privacy, insufficient teacher training, and the absence of standardized curriculum frameworks.

### 4.1 Implications for curriculum design in medical education

Several studies have demonstrated the positive impact of AI and XR technologies on student skills development and engagement (35). They used ChatGPT to simulate interactions between radiologists and patients, improving communication strategies during MRI scans (36). They showed that veterinary students benefited from AI-generated clinical cases and standardized patients, which facilitated communication training through immersive and contextualized experiences.

Other interventions, such as the study (39), integrated VASN augmented surgical navigation into endoscopic sinus surgery training, demonstrating improvements in technical performance and confidence in the procedure (40). They implemented virtual reality simulations to improve motivational interviewing and behavioral health counseling skills in pediatric residents (41). They introduced a structured AI module focused on evidence-based medicine and clinical integration, which improved students' conceptual and applied understanding. Their approach, based on reverse design and modular instruction, illustrates how AI literacy can be taught even to students with no prior technical knowledge (43). They went further by involving students in the design of AI-based clinical decision support systems, fostering critical thinking and digital leadership.

These findings point to several directions for advancing medical education with AI and XR. Interventions should be aligned with existing competency-based frameworks by mapping AI- and XR-enhanced activities to specific knowledge, skills, and professional behaviors, rather than treating them as isolated add-ons. In many of the most promising examples, AI or XR is embedded within longitudinal learning sequences, such as combining introductory modules on AI literacy with progressively more complex simulated cases and opportunities for supervised decision-making. Immersive simulations and AI-driven feedback can be directed toward competencies that are difficult to practice in routine clinical placements, including communication in high-stakes scenarios, management of rare events, and interprofessional collaboration. Several studies also highlight the value of involving students as co-designers of AI tools and learning activities, which may support digital professionalism, critical appraisal of algorithms, and more responsible adoption of these technologies in future clinical practice.

### 4.2 Methodological considerations and quality of evidence

The formal quality appraisal confirmed substantial methodological heterogeneity across the 13 included studies, both in research design and in the assessment tools employed. This diversity provides a broad overview of approaches to AI and XR in medical education but also limits comparability and constrains the strength of inferences that can be drawn from the available evidence.

A large proportion of studies relied on observational or descriptive designs, such as cross-sectional surveys (34, 35, 38) and uncontrolled educational evaluations (43, 44). These designs are useful for exploring initial perceptions, feasibility, and acceptance of new technologies, yet their capacity to establish causal relationships or accurately quantify educational impact is limited. The absence of control groups in many of these investigations, together with a frequent reliance on subjective measures—such as self-administered questionnaires or satisfaction surveys—reduces the robustness of the findings.

A smaller subset of studies adopted more rigorous designs, including randomized controlled trials (40), prospective trials (39), and educational innovations with systematic performance evaluation (41). These approaches offer comparatively stronger evidence and allow more credible inferences about the effects of AI- and XR-based interventions on clinical learning. Nevertheless, even within this group, the quality of the evidence is often constrained by small sample sizes, single-centre settings, and restricted follow-up periods. Long-term assessments of knowledge retention, skills transfer to real clinical environments, and sustained behavioural change were rarely reported, and outcome measures were not always based on validated instruments.

Another methodological consideration is the diversity of technologies and implementations evaluated. Some studies focused on virtual reality simulations (39, 40), others on conversational AI tools (35, 36) or e-learning platforms (44, 45). Across these contexts, limited faculty training, inadequate financial and technical resources, and logistical barriers were frequently highlighted. These constraints not only influence the success of implementation but also impact study design, sample recruitment, and the quality of data collection.

These features indicate that the current evidence base should be interpreted as preliminary and largely hypothesis-generating rather than definitive. While signals of benefit for AI and XR in medical and health-professions education are consistent across studies, the overall certainty of the evidence is low to moderate at best. Stronger, more standardized methodological approaches will be required to determine the true magnitude and generalizability of the educational effects attributed to these technologies.

### 4.3 Comparative with other studies

Table 6, a comparison of this review with existing literature, reveals convergences and differences in thematic focus, methodological orientation, and conceptual contributions to understanding how digital technologies—particularly AI and XR—are transforming medical education. While several previous reviews have addressed the integration of emerging technologies in medical or health-related training, this review synthesizes empirical studies across diverse clinical settings, educational modalities, and geographical contexts, offering a more comprehensive and integrative perspective.

**Table 6:** Reviews on AI, XR and digital technologies in medical education.

Reference	Central theme/objective	Main variables or constructs	Methodological orientation	Comparative notes/comments
Mese et al. (47)	Investigates how AI (especially ChatGPT) integrates with e-learning to support radiology education and proposes combining AI with expert-curated resources to improve accuracy and reliability.	ChatGPT, e-learning platforms, radiology curricula, student assessment, ethical concerns (bias, data accuracy).	Scoping review using multiple databases.	Focuses on radiology; emphasises integration of generative AI with traditional e-learning; raises concerns about accuracy and bias, echoing ethical considerations in the user's review.
Singaram et al. (48)	Assesses digital feedback tools (web platforms, apps, virtual reality and artificial intelligence) used in clinical education and identifies facilitators and barriers to adoption.	Digital tools for feedback, convenience, personalized feedback, student perceptions, barriers (technical constraints, data security).	Scoping review following Joanna Briggs Institute guidelines.	Focuses on feedback processes; highlights VR and AI for real-time feedback; emphasises geographical research imbalance and privacy concerns; complements user's review by addressing feedback dimension.
Lang et al. (17)	Explores how augmented, virtual and mixed reality (collectively extended reality, XR) provide immersive and collaborative learning in radiology, from anatomy teaching to image-guided interventions.	XR technologies (AR, VR, MR), collaborative virtual reading rooms, image analysis, procedural training, future AI integration.	Narrative/conceptual review.	Highlights XR benefits and challenges (technological, economic, ergonomic); anticipates AI-driven personalized learning; aligns closely with the AI/XR synergy emphasised in the user's review.
Awuah et al. (49)	Reviews the integration of AI, machine learning and deep learning into neurosurgical training and simulation, highlighting improvements in patient outcomes and decision-making.	AI, ML, DL, neurosurgical simulation, diagnostic and prognostic outcomes, decision-making.	Narrative literature review.	Focuses on neurosurgical specialty; emphasises AI-assisted simulation across pre-, intra- and postoperative stages; aligns with the user's emphasis on AI improving skills and patient care.
Zavala-Calahorrano et al. (50)	Systematically synthesizes literature on medical technology, AI and ChatGPT across diagnostics, treatment and education, identifying three categories: diagnostic/treatment innovations, medical education and public health/ethics.	VR/AR, AI applications, metaverse concepts (lifelogging, mirror-world), medtech, patient-centred care.	Systematic review using PRISMA and thematic analysis.	Broad scope; highlights VR/AR and metaverse in education; emphasises categories beyond education, providing context for the user's review.
Savage et al. (51)	Evaluates the effectiveness of seven technologies—including AI, immersive VR, desktop VR, needle guidance, robotics, AR and haptic devices—in regional anaesthesia training.	Artificial intelligence, immersive and desktop VR, augmented reality, robotics, haptic feedback, learner confidence and performance.	Systematic/scoping review with data extraction and qualitative synthesis.	Focuses on a specific procedural domain; demonstrates positive impact of technology-enhanced training but emphasises need for combined traditional and technological approaches.
St Mart et al. (52)	Discusses current AI pathways and technological advances in orthopaedics and how AI could transform surgical education and patient care.	AI algorithms, data analytics, robotic surgery, educational implications, patient outcomes.	Narrative review.	Focuses on orthopaedics; emphasises future integration of AI into operating rooms; aligns with the user's review by extending AI applications into another surgical domain.
Antoniou et al. (53)	Surveys training evolution in urolithiasis management, including high-/low-fidelity simulation, VR, AR and AI, and proposes curriculum pathways.	Simulation models, VR, AR, AI, standardized curricula, assessment and mentor-based training.	Narrative review.	Highlights balanced integration of simulation and mentor-guided training; emphasises stratified curricula; relates to user's focus on curriculum design.
Ramamurthy et al. (54)	Explores potential applications of the metaverse in healthcare, including AI, AR, VR, IoT, quantum computing and robotics, while addressing ethical and legal issues.	AI, AR, VR, IoT, metaverse platforms, patient interactions, ethical and legal considerations.	Conceptual review.	Broad healthcare focus; emphasises training and surgery among other applications; discusses ethics and data vulnerability, echoing concerns in the user's review.
Jaju et al. (55)	Integrative review of how COVID-19 reshaped anesthesiology training, highlighting safety measures, virtual education, AI models in ICUs and the importance of mental health.	Remote learning, PPE and barriers, sub-specialty adaptations, AI models, mental health, virtual examinations.	Integrative literature review.	Focuses on pandemic-driven changes; includes AI applications in ICU and virtual education; relates indirectly to the user's review by contextualizing digital transformation.
Soluk Tekkeşin et al. (56)	Reviews how digital technologies (e.g., radiology's digital leap, digital pathology) are transforming education, training and diagnostic workflows, especially in oral and maxillofacial pathology.	Digital imaging, telehealth, AI applications, workflow integration, educational adaptation.	Narrative review.	Focuses on dental and pathology domains; addresses digital disruption but less on AI/XR; offers context for broader technological transformation.
Hoogenboom et al. (57)	Reviews training programs for advanced endoscopic imaging (virtual chromoendoscopy, confocal laser endomicroscopy and volumetric laser endomicroscopy) and assesses learning curves and outcomes.	Advanced imaging techniques, didactic and web-based programs, learning curves, training outcomes, future AI assistance.	Narrative review of literature up to March 2020.	Focuses on GI endoscopy; emphasises web-based training efficacy; suggests need for standardized programs; mentions potential AI assistance.
Finocchiaro et al. (58)	Surveys technological developments in GI endoscopy simulators—from mechanical systems and mechatronic devices to animal models—and discusses emerging technologies such as AI, AR and robotics.	Simulation platforms (mechanical, mechatronic, animal-based), AI, AR, robotics, training needs.	Narrative review.	Examines simulation evolution; emphasises realism and pre-clinical practice; relates to user's review through simulation and emerging AI/AR technologies.
Kranjcevic et al. (59)	Narrates how the COVID-19 pandemic accelerated e-learning, teleconferencing and digital tools in academic medicine and outlines the transformation of educators’ roles.	E-learning, teleconferencing, digital publishing, educator roles, resilience, global outreach.	Narrative literature review.	Does not focus on AI/XR; provides contextual information on digital transformation during the pandemic.
Our study	Integration of Artificial Intelligence and Extended Reality in Medical Education	Digital technologies (AI, XR), Learning outcomes (skills, engagement, performance), Educational modalities		Synthesizes 13 empirical studies from 2019 to 2024, focusing on AI/XR in medical education. Identifies trends, barriers, and curricular implications

Some reviews have focused on specific tools or technologies, such as the use of ChatGPT in radiology training (47). While that review highlights the potential of generative AI to produce assessments and learning materials and points out risks such as bias and misinformation, it focuses exclusively on content generation rather than broader pedagogical outcomes. In contrast, the present study explores AI in relation to student engagement, clinical skill acquisition, and curriculum design, offering a systemic perspective that goes beyond a single educational tool.

Other studies have emphasized feedback mechanisms in clinical education (48), particularly the role of digital platforms, mobile applications, and virtual reality in facilitating personalized, real-time feedback. While our review acknowledges the value of interactive learning environments, it places greater emphasis on the curricular and infrastructure implications of adopting AI and XR, identifying challenges such as a lack of faculty training, limited technological infrastructure, and ethical considerations that may hinder implementation. Both reviews agree on the need for governance frameworks that ensure data privacy and the effectiveness of the tools, often in specific specialties such as radiology (17), neurosurgery (49), or anesthesiology (51). These reviews typically highlight the pedagogical advantages of XR for procedural training, spatial awareness, and simulation-based learning. Our review incorporates these findings, but expands the analysis across various disciplines, demonstrating that XR not only supports technical skills, but also fosters engagement and interactive learning in diverse clinical contexts, such as pediatrics, ophthalmology, and general medicine.

Broader thematic syntheses have also emerged. One review categorizes technological innovations in diagnosis, treatment, and education (50), including the use of the metaverse and patient-centered design. While this typology is useful for understanding the broader application of digital tools in healthcare, it lacks the curriculum specificity and empirical grounding present in our review. Similarly, reviews addressing the metaverse and telemedicine (54), or transformations during the COVID-19 pandemic (55), provide contextual information but offer little depth regarding educational implementation or learning outcomes.

A recurring theme in other reviews is the need to balance technological advances with traditional training approaches. This is particularly evident in areas such as regional anesthesia training (51) and urology education (53), where the value of mentor-led instruction remains, even as digital tools enhance procedural training. Our findings fully align with this perspective, advocating for the integration—not the replacement—of established pedagogical practices with AI and XR technologies. This hybrid approach ensures that digital innovation complements human interaction, reflective practice, and clinical reasoning.

While several reviews emphasize technical effectiveness, such as improved diagnostic accuracy or faster skill acquisition, systematically few address the curricular implications of these technologies. This study rectifies this deficiency by identifying the need for institutional policies, faculty training, and infrastructure development to support the sustainable integration of technology. Reviews focused on specialized training in orthopedics (52), dentistry (56), or gastroenterology (57, 58) provide valuable insights, but often limit their analysis to specific use cases within each discipline without extrapolating to broader curricular frameworks.

Our study differs methodologically from several previous works. While many are narrative or conceptual in nature, this review adopts a structured methodology using the PRISMA and PICO frameworks and focuses specifically on empirical educational interventions. By jointly examining AI-only, XR-only, and emerging AI–XR hybrid applications, it maps how these tools are currently used to support knowledge acquisition, skills performance, and learner engagement across different clinical disciplines. This synthesis underpins the curriculum-oriented recommendations developed in Sections 4.1 and 4.2, particularly in relation to competency-based design, digital literacy, and the conditions required for sustainable implementation.

### 4.4 Limitations

This study provides an overview of the integration of AI and XR in medical education, but several limitations should be noted. First, the methodological diversity of the included studies introduces significant variability in the quality of the research and the measurement of outcomes. Many studies used cross-sectional surveys or descriptive case studies, which, while useful for exploring feasibility and user perceptions, offer limited capacity to infer causality or assess long-term educational impact. The scarcity of randomized controlled trials and longitudinal follow-ups reduces the strength of the evidence and limits the generalizability of the observed effects to other institutions and populations.

A further limitation is the variable and often small sample size; some pilot studies included fewer than ten participants. These small groups limit the statistical power of the results and restrict the conclusions that can be drawn about the scalability and reproducibility of AI and XR interventions. In addition, a substantial proportion of the included reports were explicitly described as pilot, feasibility, or development studies. These early-stage designs were deliberately retained because AI- and XR-based interventions in medical and health-professions education remain an emerging field, and many innovations are first reported in small-scale or developmental projects. However, the preliminary nature of these studies means that effect estimates should be interpreted with caution and cannot be assumed to translate directly to large-scale curricular implementation. Specifically, several studies did not provide sufficient demographic or contextual information, such as participants' educational level or prior experience with digital tools—variables relevant for evaluating learning outcomes.

A related limitation is the lack of standardized outcome measures. The tools used to assess skill acquisition, participation, or performance were heterogeneous, ranging from subjective self-assessments to simulation success rates and machine learning rankings. This inconsistency hinders comparisons between studies and compromises the possibility of conducting meta-analyses or establishing benchmark performance indicators. Additionally, many reports did not provide complete descriptive statistics or effect size estimates, which further limits the interpretability of the findings and hampers direct comparison across interventions.

Technological and institutional barriers also limited the scope of many interventions. Challenges such as insufficient digital infrastructure, limited teacher training, high implementation costs, and resistance to technological change were frequently reported. These factors not only affect the feasibility of large-scale AI/XR implementation but also influence study design, often limiting the depth or rigor of intervention evaluations. A further limitation is the heterogeneity with which the technological components themselves were reported. Some articles provide detailed descriptions of algorithms, platforms, and implementation workflows, whereas others focus mainly on curricular integration or learner outcomes and describe the underlying technology only in broad terms. This inconsistency reduces the granularity with which specific AI and XR tools can be compared across studies.

This review also primarily identified research reporting positive outcomes or improvements in learning, with fewer studies critically examining implementation failures or neutral effects. Current literature may therefore overestimate the effectiveness of these technologies in educational settings; while emerging data are promising, caution is advised when interpreting the results. More robust, comparative, and longitudinal research is needed to validate the educational value of AI and XR in diverse medical training contexts.

## 5 Conclusion

The current evidence base on artificial intelligence and extended reality in medical and health-professions education is still limited and methodologically heterogeneous. Most of the 13 included studies were small, single-center evaluations with short follow-up and predominantly observational or descriptive designs, using non-standardized outcome measures and rarely examining transfer to real clinical practice. These features substantially weaken the strength and generalizability of the available evidence.

Within this context, reported benefits for knowledge, skills, performance, and engagement should be regarded as promising but preliminary signals rather than definitive proof of superiority over high-quality conventional instruction. Only a minority of interventions to date have implemented fully integrated AI–XR ecosystems; most studies evaluated AI-only or XR-only approaches. Claims about the added value of combining AI with XR must therefore remain cautious and are currently supported by a very small empirical base, further constrained by persistent implementation barriers such as costs, technical reliability, data protection, and insufficient faculty development.

Future research should prioritize rigorously designed studies with adequate sample sizes, validated and comparable outcomes, and longitudinal follow-up, explicitly distinguishing between AI-only, XR-only, and fully integrated AI–XR interventions. In parallel, efforts to strengthen digital literacy, ethical and regulatory awareness, and faculty preparation within competency-based curricula will be essential to determine whether these technologies can deliver sustainable and equitable improvements in medical education.

## Data Availability

The original contributions presented in the study are included in the article/Supplementary Material, further inquiries can be directed to the corresponding author.
